# HMGB1 Deficiency Occurs in a Broad Range of Human Cancers and Is Often Associated with Unfavorable Tumor Phenotype

**DOI:** 10.3390/diagnostics15151974

**Published:** 2025-08-06

**Authors:** Viktoria Chirico, Hena Sharifi, Maria Christina Tsourlakis, Seyma Büyücek, Clara Marie von Bargen, Katharina Möller, Florian Lutz, David Dum, Martina Kluth, Claudia Hube-Magg, Georgia Makrypidi-Fraune, Piero Caneve, Maximilian Lennartz, Morton Freytag, Sebastian Dwertmann Rico, Simon Kind, Viktor Reiswich, Eike Burandt, Till S. Clauditz, Patrick Lebok, Christoph Fraune, Till Krech, Sarah Minner, Andreas H. Marx, Waldemar Wilczak, Ronald Simon, Guido Sauter, Stefan Steurer, Kristina Jansen

**Affiliations:** 1 Institute of Pathology, University Medical Center Hamburg-Eppendorf, 20246 Hamburg, Germany; v.chirico@uke.de (V.C.); hena.sha@hotmail.com (H.S.); mtsourlakis@uke.de (M.C.T.); s.bueyuecek@uke.de (S.B.); c.von-bargen@uke.de (C.M.v.B.); ka.moeller@uke.de (K.M.); f.lutz@uke.de (F.L.); d.dum@uke.de (D.D.); m.kluth@uke.de (M.K.); c.hube@uke.de (C.H.-M.); g.makrypidi@uke.de (G.M.-F.); p.caneve@uke.de (P.C.); m.lennartz@uke.de (M.L.); m.freytag@uke.de (M.F.); s.dwertmann-rico@uke.de (S.D.R.); s.kind@uke.de (S.K.); v.reiswich@uke.de (V.R.); e.burandt@uke.de (E.B.); t.clauditz@uke.de (T.S.C.); p.lebok@uke.de (P.L.); c.fraune@uke.de (C.F.); t.krech@uke.de (T.K.); s.minner@uke.de (S.M.); w.wilczak@uke.de (W.W.); g.sauter@uke.de (G.S.); s.steurer@uke.de (S.S.); 2 Institute of Pathology, Clinical Center Osnabrück, 49074 Osnabrück, Germany; 3 Department of Pathology, Academic Hospital Fürth, 90766 Fürth, Germany; andreas.marx@klinikum-fuerth.de; 4 General, Visceral and Thoracic Surgery Department and Clinic, University Medical Center Hamburg-Eppendorf, 20246 Hamburg, Germany; k.jansen@uke.de

**Keywords:** HMGB1 deficiency, human cancers, immunohistochemistry, tissue microarray, tumor phenotype

## Abstract

**Background/Objectives**: Aberrant expression of high-mobility group protein B1 (HMGB1) has been linked to cancer development and progression. **Methods**: To better comprehend the role of HMGB1 expression in cancer, a tissue microarray containing 14,966 samples from 134 different tumor entities and 608 samples of 76 different normal tissue types was analyzed by immunohistochemistry. **Results**: Strong HMGB1 staining occurred in almost all normal cell types and in most cancers. Of 11,808 evaluable cancers, only 7.8% showed complete absence of HMGB1 staining (HMGB1 deficiency) while 9.9% showed 1+, 25.0% showed 2+, and 57.2% showed 3+ HMGB1 positivity. Absence of HMGB1 staining mostly occurred in pheochromocytoma (90.0%), seminoma (72.4%), gastrointestinal stromal tumor (28.6%), adrenal cortical carcinoma (25.0%), and Hodgkin’s lymphoma (25.0%). Low HMGB1 staining was linked to poor histologic grade (*p* < 0.0001), advanced pT stage (*p* < 0.0001), high UICC stage (*p* < 0.0001), and distant metastasis (*p* = 0.0413) in clear cell renal cell carcinoma, invasive tumor growth in urothelial carcinoma (pTa vs. pT2–4, *p* < 0.0001), mismatch repair deficiency (*p* = 0.0167) in colorectal cancers, and advanced pT stage in invasive breast carcinoma of no special type (*p* = 0.0038). Strong HMGB1 staining was linked to nodal metastases in high-grade serous ovarian carcinomas (*p* = 0.0213) and colorectal adenocarcinomas (*p* = 0.0137), as well as to poor histological grade in squamous cell carcinomas (*p* = 0.0010). **Conclusions**: HMGB1 deficiency and reduced HMGB1 expression occur in a broad range of different tumor entities. Low rather than strong HMGB1 staining is often linked to an aggressive tumor phenotype. Whether HMGB1 deficiency renders cells susceptible to specific drugs remains to be determined.

## 1. Introduction

High-mobility group protein B1 (HMGB1) is the second most common protein after histone in the nucleus of most human cell types. Like all other members of the non-histone chromosomal high-mobility group protein family, HMGB1 is a chromatin-associated protein. It has a role in the maintenance of nucleosome structure, regulation of DNA replication, transcription control, and DNA repair through a specific binding to damaged DNA. HMGB1 increases the binding affinity of many transcription factors, such as p53, Rb, NF-κB, and estrogen receptor, to their target DNA sequences [1]. Critical additional functions of HMGB1 require an extra-nuclear location of the protein. In the cytoplasm, HMGB1 participates in the control of autophagy and apoptosis [2,3]. After release to the extracellular space, HMGB1 acts as a damage-associated molecular pattern molecule (DAMP) that critically mediates inflammation and immune responses in various conditions, including sepsis, atherosclerosis, arthritis, neurodegeneration, meningitis, and cancer. Therapeutic options to regulate HMGB1 in preclinical models are being evaluated in the field of non-neoplastic diseases (sepsis, inflammation) [4], as well as in cancer [5,6,7,8,9].

The role of HMGB1 in cancer is complex and not well understood. Different mechanisms activated by HMGB1 contribute to critical cancer cell traits such as autophagy, immunogenic cell death (ICD), release of cytokines and chemokines, angiogenesis, and migration [10,11,12]. Both increased and reduced levels of HMGB1 in cancer cells have been found to be linked to aggressive cancer phenotype, and it was proposed that these different roles may depend on the tissue/cell types involved [13].

In case of overexpression, several pivotal transcription factors, such as p53, retinoblastoma (RB) proteins, and NF-κB family members, can enhance their oncogenic activities through direct interactions with HMGB1 [14,15,16]. Reduced levels of expression or activity of HMGB1 in cancer cells may result in an unfavorable disease course due to increased levels of genomic instability [17,18]. The fact that HMGB1 is expressed not only in cancer cells but also in virtually all cell types of the tumor microenvironment adds further complexity and constitutes a challenge for studying HMGB1 in cancer in vivo. Although immunohistochemistry (IHC) enables a cell type and cell compartment-specific evaluation of HMGB1 protein, the number of previous studies is limited, and the obtained data are difficult to compile because of different antibodies, staining protocols, and evaluation criteria that were used in these studies.

We hypothesized that HMGB1 alterations may correlate with more aggressive clinicopathological features in different tumor types. Accordingly, more than 14,000 tissue samples from 134 different tumor types and subtypes, as well as 76 non-neoplastic tissues, were evaluated by IHC in a tissue microarray (TMA) format. Results were compared with histological parameters of malignancy, clinical outcome in some tumor entities, as well as with previously collected data on the immune cell tumor microenvironment.

## 2. Materials and Methods

### 2.1. Tissue Microarrays (TMAs)

Our normal tissue TMA was composed of 8 samples from 8 different donors for each of 76 different normal tissue types (608 samples on one slide). The cancer TMAs contained a total of 14,966 primary tumors from 134 tumor types and subtypes. Detailed histopathological and molecular data were available for cancers of the kidney (n = 1757), urinary bladder (n = 829), colorectum (n = 2351), pancreas (n = 598), stomach (n = 327), liver (n = 301), ovary (n = 524), and the thyroid gland (n = 518). Data on pT, pN, grade, and HPV status were available from 902 squamous cell carcinomas of 11 different sites of origin. The composition of both normal and cancer TMAs is described in detail in Section 3. All samples were obtained from the archives of the pathology institutes in Hamburg, Osnabrück, and Fürth (University Medical Center Hamburg-Eppendorf, Clinical Center Osnabrück, Academic Hospital Fürth, Germany). Tissues were fixed in 4% buffered formalin and then embedded in paraffin. TMA tissue spot diameter was 0.6 mm. The use of archived remnants of diagnostic tissues for the manufacturing of TMAs and their analysis for research purposes, as well as patient data analysis, is permitted in accordance with local laws (HmbKHG, §12,1) and has been approved by the local ethics committee (Ethics commission Hamburg, WF-049/09). All work has been carried out in compliance with the Helsinki Declaration.

### 2.2. Immunohistochemistry

Freshly cut TMA sections were immunostained on one day and in one experiment. Slides were deparaffinized with xylol, rehydrated through a graded alcohol series, and exposed to heat-induced antigen retrieval for 5 min in an autoclave at 121 °C in pH 7.8 Tris-EDTA-Citrate (TEC) buffer. Endogenous peroxidase activity was blocked with Dako REAL Peroxidase-Blocking Solution (Agilent Technologies, Santa Clara, CA, USA; #S2023) for 10 min. Primary antibody specific for HMGB1 (recombinant rabbit monoclonal, clone HMV317, cat. #6559-317-01, MS Validated Antibodies GmbH, Hamburg, Germany) was applied at 37 °C for 60 min at a dilution of 1:150. This immunostaining protocol was developed using a small test TMA containing negative (spermatids in the testis) and positive (colon mucosa) control tissues. The normal tissue TMA (NTA) was also analyzed with an independent antibody against HMGB1 (EPR3507, Abcam, Cambridge, U.K., cat. #ab79823, dilution 1:3600) to validate the specificity of HMV317 by comparing the staining patterns of both antibodies as suggested by the International Working Group for Antibody Validation (IWGAV) [19]. According to the IWGA, specificity can be assumed when two independent antibodies show identical staining, while all staining observed only with one but not with the other antibody must be considered antibody-specific cross-reactivity. The NTA, with its broad range of human healthy tissues, is optimally suited to detect antibody-specific cross-reactivity as it represents virtually all human proteins. Examples for the comparison of the two antibodies are provided in Supplementary Figure S1. Bound antibody was then visualized using the Dako REAL EnVision Detection System Peroxidase/DAB+, Rabbit/Mouse kit (Agilent Technologies, Santa Clara, CA, USA; #K5007) according to the manufacturer’s directions. The sections were counterstained with hemalaun. One experienced pathologist (V.C.) analyzed all tumor TMA sections according to an established scoring system [20]. In brief, the staining intensity of tumor cells was semi-quantitatively recorded as 0, 1+, 2+, or 3+. Presence of unequivocal HMGB1 positivity in stroma cells was particularly required to classify a tumor as “0” (HMGB1 completely negative, HMGB1 deficiency). Tumors with the absence of HMGB1 staining in both tumor and stroma cells were categorized as “non-informative”. This scoring scheme was selected to allow for unequivocal identification of tumors with HMGB1 deficiency, for statistical comparison between tumors with and without HMGB1 deficiency, and between tumors with low (1+), high (3+), or intermediate (2+) HMGB1 expression Supplementary Figure S2.

### 2.3. Statistics

Statistical calculations were performed with JMP^®^ version 18 software (SAS^®^, Cary, NC, USA). Contingency tables and the chi^2^-test were performed to search for associations between HMGB1 immunostaining and tumor phenotype.

## 3. Results

### 3.1. Technical Issues

A total of 11,808 (78.9%) of 14,966 tumor samples were interpretable in our TMA analysis. Non-interpretable samples demonstrated a lack of unequivocal tumor cells, a lack of the entire tissue spot, or a lack of staining in neoplastic and normal cells. A sufficient number of samples (≥4) of each normal tissue type was evaluable.

### 3.2. HMGB1 in Normal Tissues

**Figure 1:**
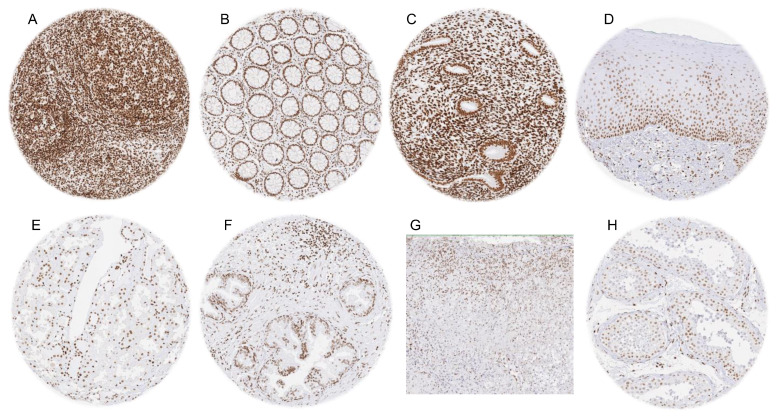
HMGB1 immunostaining of normal tissues. The panels show a strong nuclear staining in lymphocytes, epithelial and stroma cells of the tonsil (**A**), as well as in epithelial and stromal cells of the colon (**B**) and the endometrium (**C**) while the HMGB1 staining intensity decreased from the basal towards the superficial cell layers in squamous epithelium of the oral mucosa (**D**). A less intense nuclear staining was seen in proximal tubules of the kidney (**E**) and in acinar cells of the prostate gland (**F**). HMGB1 staining was absent or only weak in medullary cells of the adrenal gland (**G**), and in maturing cells of the spermatogenesis in the testis (**H**).

A distinct, and usually strong, nuclear HMGB1 staining was observed in all tissues and in the vast majority of cell types. HMGB1 staining was particularly strong in lymphocytes and other cells of the immune system, all cells of the hematopoietic system, epithelial cells of the gastrointestinal tract, pneumocytes, epithelial cells of the fallopian tube, breast epithelial cells, epithelial and stromal cells of the proliferating endometrium, ovarian stroma cells, epithelial cells of the parathyroid gland, and in granule cells of the cerebrum. In squamous epithelium, HMGB1 staining was strong in the basal and suprabasal cell layers, but the staining intensity continuously decreased towards the superficial cell layers, where the staining sometimes was faint. Nuclear HMGB1 labeling was also rather weak in central areas of corpuscles of Hassall’s of the thymus, hepatocytes, Brunner glands, proximal tubuli of the kidney, acinar cells of the prostate (while it was markedly stronger in basal cells), and in corpus luteum cells of the ovary. Nuclear HMGB1 staining was—under the selected conditions—faint or absent in heart muscle, epithelial cells of the adenohypophysis, pituicytes of the neurohypophysis, and in maturing germ cells of the testis (spermatocytes, spermatids). HMGB1 staining was distinct in cortical cells but only weak or even absent in medullary cells. Representative images are shown in Figure 1. All these stainings, including the distinction between low- and high-expressing cells, were obtained with both the HMV317 and the EPR3507 antibody (Supplementary Figure S1).

### 3.3. HMGB1 in Tumor Tissues

A nuclear HMGB1 staining was seen in all cells of the vast majority of tumors. Of 11,808 evaluable tumors, only 921 (7.8%) showed a complete lack of HMGB1 expression (staining intensity “0”, HMGB1 deficiency) while 1172 (9.9%) showed 1+, 2956 (25.0%) showed 2+, and 6759 (57.2%) showed 3+ HMGB1 positivity (Table 1).

**Figure 2:**
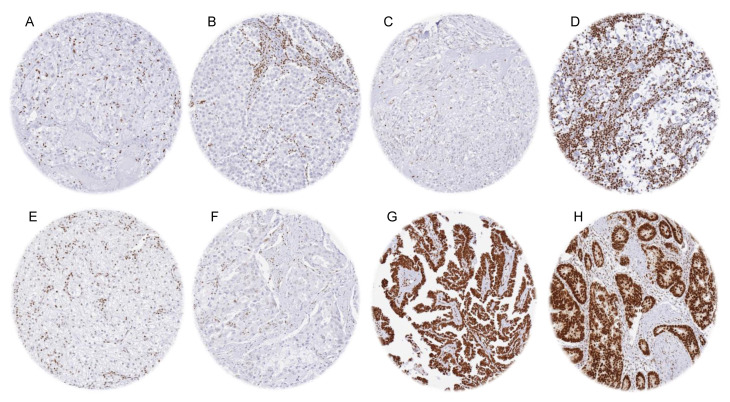
HMGB1 immunostaining in cancer. The panels show a complete absence of nuclear HMGB1 staining in all tumor cells but retained staining in non-neoplastic cells of a phaeochromocytoma (**A**), a seminoma (**B**), a gastrointestinal stroma tumor (**C**), a Hodgkin’s lymphoma (**D**), a clear cell renal cell carcinoma (**E**), and a prostatic adenocarcinoma (**F**). Examples of HMGB1-positive tumors include a serous adenocarcinoma of the ovary (**G**) and an adenocarcinoma of the colon (**H**).

HMGB1 deficiency most commonly occurred in pheochromocytoma (90.0%), seminoma (72.4%), gastrointestinal stromal tumor (28.6%), adrenal cortical carcinoma (25.0%), Hodgkin’s lymphoma (25.0%), Leydig cell tumor of the testis (16.7%), prostatic adenocarcinoma (8.9–16.4%), renal cell tumors (8.4–16.2%), yolk sac tumor of the testis (15.4%), paraganglioma (12.5%), neuroendocrine tumor of the lung (11.1%), adrenocortical adenoma (10.8%), epithelioid mesothelioma (10.7%), and in clear cell carcinoma of the ovary (9.8%). HMGB1 deficiency in <10% was seen in 52 other tumor categories. Representative images are shown in Figure 2, and other tumor entities are in Supplementary Figure S3.

**Figure 3:**
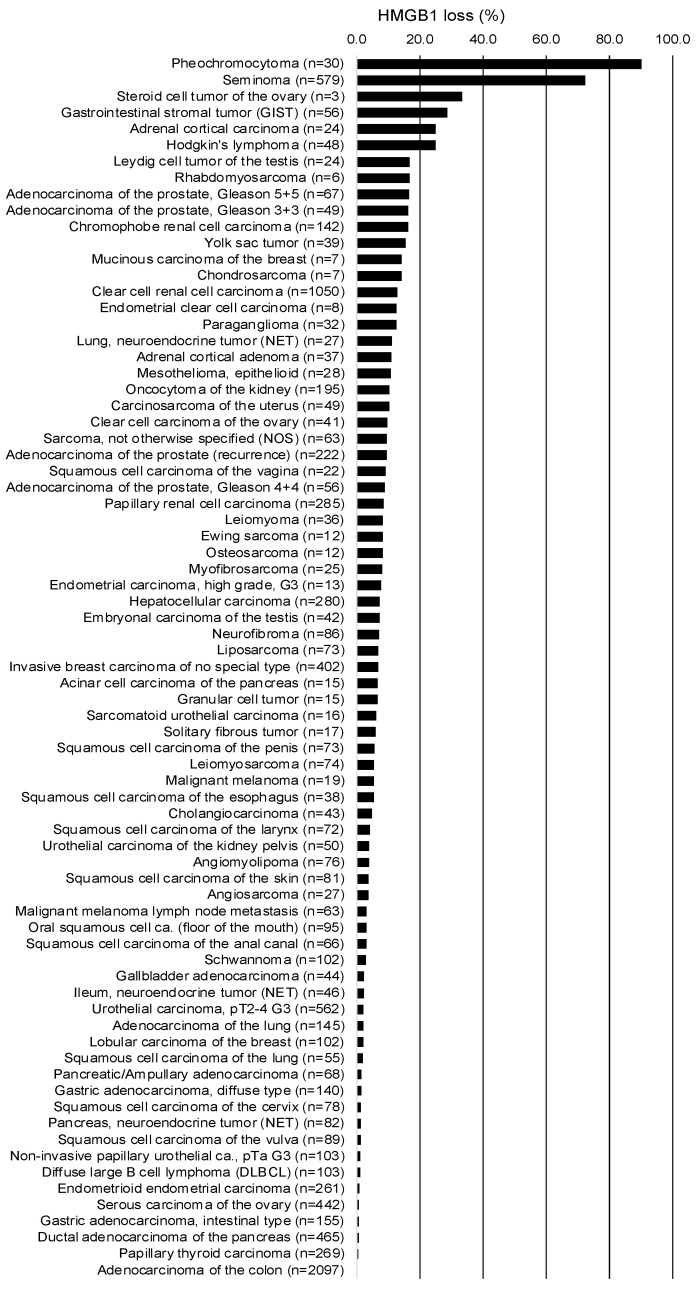
Ranking of human tumor types according to the frequency of HMGB1 expression loss.

A ranking order of tumors according to their rate of HMGB1 deficiency is given in Figure 3.

The comparison with clinico-pathological parameters of a malignant tumor phenotype revealed significant associations between altered HMGB1 expression and aggressive tumor phenotype in multiple tumor entities (Table 2).

Weak HMGB1 immunostaining was linked to poor histological grade (*p* < 0.0001), advanced pT stage (*p* < 0.0001), high UICC stage (*p* < 0.0001), nodal metastases (*p* = 0.0108) and distant metastasis (*p* = 0.0413) in clear cell renal cell carcinomas (ccRCC), invasive tumor growth in urothelial carcinoma (pTa vs. pT2–4, *p* < 0.0001), mismatch repair deficiency (*p* = 0.0167) in colorectal cancers, and advanced pT stage in invasive breast carcinomas of no special type (NST; *p* = 0.0038). Strong HMGB1 immunostaining was linked to nodal metastases in high-grade serous ovarian carcinomas (*p* = 0.0213) and colorectal cancers (*p* = 0.0137), as well as to high histological grade in a combined analysis of squamous cell carcinomas originating from 11 different organs (*p* = 0.0010). HMGB1 immunostaining was unrelated to parameters of cancer aggressiveness in pancreatic and gastric adenocarcinomas, seminomas, as well as in endometrioid endometrial carcinomas.

## 4. Discussion

The successful IHC analysis of 11,808 tumors from 134 different entities, together with a broad range of different normal tissues, provides a comprehensive overview of the pattern of HMGB1 expression in human tumors. The approximately 3000 non-analyzable TMA spots correspond to the typical rate of approximately 10–20% non-interpretable tissue spots in TMA studies [21,22]. Since the causes (missing spots or absence of tumor cells) can affect any spot of any tumor type at random, a general bias for the study results can be excluded. That a complete loss of HMGB1 expression (HMGB1 deficiency) can be found in at least one individual tumor from more than 75 different tumor categories represents a major finding of this study. The ranking list of tumors according to their rate of HMGB1 deficiency (Figure 3) provides probably the most comprehensive view of this alteration available to date, although the accuracy of our data is still limited by small numbers in some tumor categories. It is of note that seminoma and pheochromocytoma, the tumors with—by far—the highest rate of cases with HMGB1 deficiency, are derived from HMGB1-negative cell types. In these tumors lack of HMGB1 expression may represent a ”normal” status, while a lack of HMGB1 protein will indicate “HMGB1 deficiency” in most or all other tumor entities. In line with our data, others described HMGB1 deficiency in tumor cells to occur in subsets of colorectal [23] and ovarian cancers [24]. The large size of several of our tumor cohorts enabled us to address the clinical relevance of HMGB1 deficiency in various cancer types. The significant associations between HMGB1 deficiency and dismal tumor phenotype in ccRCC, urothelial carcinoma, and invasive breast carcinomas of no special type (NST) argue for low HMGB1 expression levels, going along with aggressive cancer behavior at least in certain cancer entities. Significant associations between HMGB1 deficiency in tumor cells and unfavorable tumor features have also been previously reported in pancreatic cancer [25] and endometrial cancer [26].

The molecular mechanisms resulting in reduced or lost HMGB1 expression and increased aggressiveness of HMGB1-depleted cancer cells are not fully understood. It has been shown, however, that genetic deletions [27], promoter hypermethylation [28], and microRNA-mediated suppression [29] can contribute to HMGB1 loss, and that HMGB1 deficiency can affect both tumor cell biology and tumor microenvironment. In line with an important role of HMGB1 for maintenance of genomic integrity, mouse fibroblasts lacking HMGB1 suffer from pronounced chromosomal instability and display higher rates of damage after UV irradiation than wildtype controls [17]. In pancreatic models, HMGB1 loss leads to oxidative DNA damage, chromosomal rearrangements, and telomere abnormalities, resulting in inflammatory nucleosome release and the propagation of KRAS-driven tumorigenesis [25]. Moreover, it has been shown that HMGB1-deficient tumors resist DNA-alkylating therapies and have an impaired ability to recruit immune cells into the treated tumor tissue, resulting in impaired apoptosis and improved cancer cell survival [30]. Although it cannot be excluded that loss of HMGB1 in cells of advanced tumors does not represent a functionally significant modification but just reflects tumor cell dedifferentiation, which typically parallels cancer progression, it appears counterintuitive that loss of the most abundant histone protein in cancer cells remains without a notable functional effect.

The complete loss of a protein with an important cellular function in tumor cells may offer therapeutic options targeting proteins from associated signaling pathways, such as DNA damage or innate immune response. In the case of HMGB1 deficiency, potential strategies have been proposed, although clinical trials are lacking to date. Since loss of HMGB1 in cancer cells leads to impaired DNA repair in vitro and in vivo, it has been suggested that HMGB1 deficiency may increase sensitivity to alkylating agents or ionizing radiation [31,32]. Moreover, the combination of chemotherapy and Toll-like receptor 4 (TLR4) agonists has been proposed as a potentially promising approach. Because HMGB1-deficient cancers may have limited capacity to recruit inflammatory cells through secretion of HMGB1, it was speculated that TLR4 agonists may be able to substitute HMGB1 in its role of activating T cells by binding to TLR4. One earlier study had shown that the use of TRL4 agonists promoted immunogenic cell death and may have helped to overcome the resistance observed in HMGB1-deficient tumors [33]. Irrespective of a possible type of action, our ranking order of tumors according to their rate of HMGB1 deficiency describes the tumor entities that might benefit most from potential future drugs for targeting HMGB1-deficient tumors.

Several previous IHC studies have reported associations between high HMGB1 immunostaining and unfavorable tumor phenotype or poor prognosis in non-small-cell lung cancer [34], cervical cancer [35], squamous cell carcinoma of the esophagus [36], squamous cell carcinoma of the larynx [37], urothelial carcinoma of the bladder [38], breast cancer [39], as well as colorectal [27] and gastric adenocarcinoma [40,41,42]. Our data revealed only comparatively weak associations between increased HMGB1 expression and unfavorable tumor features in serous ovarian carcinoma, colorectal cancers, and squamous cell carcinomas. It is of note, however, that our IHC protocol was designed to be highly sensitive by increasing the antibody concentration to the highest possible level before disturbing background staining occurred. Such a protocol ensures that even very low expression is reliably detected but also results in a loss of discrimination at high expression levels. As a result, the group of 3+ cases was quite large in most of our tumor categories. It cannot be excluded that a markedly less sensitive protocol would have distinguished a subgroup of tumors with a particularly high HMGB1 expression within our group 3+ positive tumors, and that these tumors could be characterized by increased aggressiveness. Moreover, due to low numbers of cases, associations with tumor phenotype could not be evaluated in this study for several cancer types (non-small-cell lung cancer, cervical cancer, gastric adenocarcinoma) for which an oncogenic role of HMGB1 has previously been described [27,36,37,38,39,40,41,42]. An oncogenic role of HMGB1—at least in specific tumor types—can therefore not be excluded.

Because of the ubiquitous expression of HMGB1 in virtually all cell types of the tumor stroma, IHC is the optimal method for assessing the HMGB1 status of a tumor. Automated image analysis tools could even enhance IHC scoring in specific tumor types, but algorithms that would enable the distinction between tumor and non-neoplastic cells across a broad range of different tumor types are currently lacking.

Considering the high variability of published IHC data on HMGB1, a particular emphasis was placed on the validation of our IHC assay. The International Working Group for Antibody Validation (IWGAV) has proposed that an acceptable antibody validation for IHC on formalin-fixed tissues must include either a comparison of IHC findings with a second antibody for the same target or a comparison with another independent method for expression analysis [19]. Because a comparison with data obtained by a method using disaggregated tissue is inappropriate in the case of ubiquitously expressed proteins, a thorough comparison with an independent second antibody was performed on consecutive TMA sections containing samples from 76 different normal tissue categories. The specificity of our assay was confirmed because identical staining patterns were observed with both HMV317 and EPR3507 antibodies, particularly in these tissues, where the staining displayed a variability between different cell types. These included a decrease in the staining intensity from basal to superficial cell layers in squamous epithelium and the absence of HMGB1 in maturing spermatids and spermatozoa of the testis and in medullary cells of the adrenal gland. A very broad range of different tissues for antibody validation increases the likelihood of detecting antibody cross-reactivities because virtually all proteins occurring in normal cells of adult humans are subjected to the validation experiment.

In summary, our data show that HMGB1 deficiency and reduced HMGB1 expression occur in a broad range of different tumor entities and that low rather than high HMGB1 expression is often linked to an aggressive tumor phenotype, thereby bridging the gap between basic molecular insights and clinical applications in cancer. If drugs became available that target HMGB1 deficiency, immunohistochemical testing would be suitable to identify patients who could potentially benefit from such therapies. However, it remains to be clarified whether HMGB1 deficiency makes the cells susceptible to certain drugs, whether HMGB1 deficiency can be used as a prognostic marker, and which thres-hold values are clinically applicable to define an immunohistochemical HMGB1 deficiency. In view of the retrospective nature and the limited number of cases of some tumor types, further studies for prospective validation in patient cohorts with follow-up data would be desirable.

## Figures and Tables

**Table 1:** HMGB1 immunostaining in human tumors. int. = interpretable.

		On TMA (n)	HMGB1 Immunostaining				
	Tumor Entity		int. (n)	0 (%)	1+ (%)	2+ (%)	3+ (%)
Tumors of the skin	Basal cell carcinoma of the skin	41	26	0.0	0.0	0.0	100.0
	Squamous cell carcinoma of the skin	95	81	3.7	11.1	50.6	34.6
	Malignant melanoma	19	19	5.3	10.5	47.4	36.8
	Malignant melanoma lymph node metastasis	86	63	3.2	6.3	20.6	69.8
	Merkel cell carcinoma	2	2	0.0	50.0	50.0	0.0
Tumors of the head and neck	Squamous cell carcinoma of the larynx	109	72	4.2	16.7	22.2	56.9
	Squamous cell carcinoma of the pharynx	60	47	0.0	14.9	31.9	53.2
	Oral squamous cell carcinoma (floor of the mouth)	130	95	3.2	13.7	35.8	47.4
	Pleomorphic adenoma of the parotid gland	50	21	0.0	9.5	4.8	85.7
	Warthin tumor of the parotid gland	49	32	0.0	0.0	15.6	84.4
	Basal cell adenoma of the salivary gland	15	9	0.0	0.0	22.2	77.8
Tumors of the lung, pleura, and thymus	Adenocarcinoma of the lung	196	145	2.1	5.5	38.6	53.8
	Squamous cell carcinoma of the lung	80	55	1.8	16.4	30.9	50.9
	Mesothelioma, epithelioid	40	28	10.7	25.0	25.0	39.3
	Mesothelioma, biphasic	29	15	0.0	26.7	33.3	40.0
	Thymoma	29	18	0.0	11.1	11.1	77.8
	Lung, neuroendocrine tumor (NET)	29	27	11.1	3.7	18.5	66.7
Tumors of the female genital tract	Squamous cell carcinoma of the vagina	30	22	9.1	9.1	31.8	50.0
	Squamous cell carcinoma of the vulva	107	89	1.1	16.9	24.7	57.3
	Squamous cell carcinoma of the cervix	88	78	1.3	20.5	39.7	38.5
	Adenocarcinoma of the cervix	23	22	0.0	4.5	13.6	81.8
	Endometrioid endometrial carcinoma	288	261	0.8	10.0	39.8	49.4
	Endometrial serous carcinoma	36	31	0.0	3.2	32.3	64.5
	Carcinosarcoma of the uterus	57	49	10.2	14.3	30.6	44.9
	Endometrial carcinoma, high grade, G3	13	13	7.7	23.1	38.5	30.8
	Endometrial clear cell carcinoma	9	8	12.5	37.5	25.0	25.0
	Endometrioid carcinoma of the ovary	93	71	0.0	12.7	32.4	54.9
	Serous carcinoma of the ovary	530	442	0.7	13.1	33.9	52.3
	Mucinous carcinoma of the ovary	75	52	0.0	7.7	21.2	71.2
	Clear cell carcinoma of the ovary	51	41	9.8	17.1	34.1	39.0
	Carcinosarcoma of the ovary	47	37	0.0	2.7	24.3	73.0
	Granulosa cell tumor of the ovary	44	35	0.0	8.6	14.3	77.1
	Leydig cell tumor of the ovary	4	3	0.0	66.7	0.0	33.3
	Sertoli cell tumor of the ovary	1	1	0.0	0.0	100.0	0.0
	Sertoli Leydig cell tumor of the ovary	3	3	0.0	0.0	0.0	100.0
	Steroid cell tumor of the ovary	3	3	33.3	33.3	33.3	0.0
	Brenner tumor	32	28	0.0	25.0	7.1	67.9
Tumors of the breast	Invasive breast carcinoma of no special type	499	402	6.7	13.7	37.3	42.3
	Lobular carcinoma of the breast	150	102	2.0	13.7	40.2	44.1
	Medullary carcinoma of the breast	8	7	0.0	0.0	71.4	28.6
	Tubular carcinoma of the breast	2	1	0.0	0.0	0.0	100.0
	Mucinous carcinoma of the breast	7	7	14.3	14.3	28.6	42.9
Tumors of the digestive system	Adenomatous polyp, low-grade dysplasia	50	34	0.0	0.0	2.9	97.1
	Adenomatous polyp, high-grade dysplasia	50	45	0.0	2.2	2.2	95.6
	Adenocarcinoma of the colon	2483	2097	0.2	3.9	20.6	75.3
	Gastric adenocarcinoma, diffuse type	215	140	1.4	10.7	28.6	59.3
	Gastric adenocarcinoma, intestinal type	215	155	0.6	11.0	26.5	61.9
	Gastric adenocarcinoma, mixed type	62	45	0.0	20.0	33.3	46.7
	Adenocarcinoma of the esophagus	83	42	0.0	0.0	21.4	78.6
	Squamous cell carcinoma of the esophagus	76	38	5.3	13.2	21.1	60.5
	Squamous cell carcinoma of the anal canal	91	66	3.0	10.6	25.8	60.6
	Cholangiocarcinoma	58	43	4.7	11.6	32.6	51.2
	Gallbladder adenocarcinoma	51	44	2.3	4.5	31.8	61.4
	Gallbladder Klatskin tumor	42	35	0.0	8.6	14.3	77.1
	Hepatocellular carcinoma	312	280	7.1	14.3	33.9	44.6
	Ductal adenocarcinoma of the pancreas	659	465	0.6	7.5	34.2	57.6
	Pancreatic/Ampullary adenocarcinoma	98	68	1.5	10.3	36.8	51.5
	Acinar cell carcinoma of the pancreas	18	15	6.7	13.3	33.3	46.7
	Gastrointestinal stromal tumor (GIST)	62	56	28.6	32.1	26.8	12.5
	Appendix, neuroendocrine tumor (NET)	25	10	0.0	20.0	30.0	50.0
	Colorectal, neuroendocrine tumor (NET)	12	9	0.0	11.1	33.3	55.6
	Ileum, neuroendocrine tumor (NET)	53	46	2.2	6.5	28.3	63.0
	Pancreas, neuroendocrine tumor (NET)	101	82	1.2	4.9	31.7	62.2
	Colorectal, neuroendocrine carcinoma (NEC)	14	12	0.0	8.3	33.3	58.3
	Ileum, neuroendocrine carcinoma (NEC)	8	7	0.0	28.6	14.3	57.1
	Gallbladder, neuroendocrine carcinoma (NEC)	4	4	0.0	0.0	50.0	50.0
	Pancreas, neuroendocrine carcinoma (NEC)	14	10	0.0	10.0	50.0	40.0
Tumors of the urinary system	Non-invasive papillary urothelial ca., pTa G2 low grade	87	78	0.0	1.3	3.8	94.9
	Non-invasive papillary urothelial ca., pTa G2 high grade	80	68	0.0	0.0	4.4	95.6
	Non-invasive papillary urothelial car., pTa G3	126	103	1.0	1.9	14.6	82.5
	Urothelial carcinoma, pT2–4 G3	735	562	2.1	9.3	29.2	59.4
	Squamous cell carcinoma of the bladder	22	19	0.0	21.1	10.5	68.4
	Small cell neuroendocrine carcinoma of the bladder	5	5	0.0	0.0	0.0	100.0
	Sarcomatoid urothelial carcinoma	25	16	6.3	18.8	31.3	43.8
	Urothelial carcinoma of the kidney pelvis	62	50	4.0	0.0	16.0	80.0
	Clear cell renal cell carcinoma	1287	1050	12.7	7.7	21.9	57.7
	Papillary renal cell carcinoma	368	285	8.4	9.1	27.7	54.7
	Clear cell (tubulo) papillary renal cell carcinoma	26	18	0.0	5.6	5.6	88.9
	Chromophobe renal cell carcinoma	170	142	16.2	14.1	28.2	41.5
	Oncocytoma of the kidney	257	195	10.3	11.8	33.3	44.6
Tumors of the male genital organs	Adenocarcinoma of the prostate, Gleason 3+3	83	49	16.3	32.7	32.7	18.4
	Adenocarcinoma of the prostate, Gleason 4+4	80	56	8.9	32.1	37.5	21.4
	Adenocarcinoma of the prostate, Gleason 5+5	85	67	16.4	20.9	41.8	20.9
	Adenocarcinoma of the prostate (recurrence)	258	222	9.5	18.9	38.3	33.3
	Small cell neuroendocrine carcinoma of the prostate	2	1	0.0	0.0	0.0	100.0
	Seminoma	682	579	72.4	18.3	8.6	0.7
	Embryonal carcinoma of the testis	54	42	7.1	47.6	38.1	7.1
	Leydig cell tumor of the testis	31	24	16.7	33.3	33.3	16.7
	Sertoli cell tumor of the testis	2	2	0.0	0.0	0.0	100.0
	Sex cord stromal tumor of the testis	1	1	0.0	100.0	0.0	0.0
	Spermatocytic tumor of the testis	1	1	0.0	100.0	0.0	0.0
	Yolk sac tumor	53	39	15.4	35.9	30.8	17.9
	Teratoma	53	35	0.0	20.0	5.7	74.3
	Squamous cell carcinoma of the penis	92	73	5.5	8.2	41.1	45.2
Tumors of endocrine organs	Adenoma of the thyroid gland	63	49	0.0	2.0	20.4	77.6
	Papillary thyroid carcinoma	341	269	0.4	1.9	13.8	84.0
	Follicular thyroid carcinoma	109	56	0.0	3.6	23.2	73.2
	Medullary thyroid carcinoma	57	45	0.0	13.3	31.1	55.6
	Parathyroid gland adenoma	43	27	0.0	0.0	0.0	100.0
	Anaplastic thyroid carcinoma	19	14	0.0	28.6	50.0	21.4
	Adrenal cortical adenoma	48	37	10.8	18.9	24.3	45.9
	Adrenal cortical carcinoma	27	24	25.0	33.3	25.0	16.7
	Pheochromocytoma	51	30	90.0	10.0	0.0	0.0
Tumors of hematopoietic and lymphoid tissues	Hodgkin’s lymphoma	103	48	25.0	31.3	41.7	2.1
	Small lymphocytic lymphoma, B-cell type (B-SLL/B-CLL)	50	45	0.0	0.0	6.7	93.3
	Diffuse large B-cell lymphoma (DLBCL)	113	103	1.0	15.5	28.2	55.3
	Follicular lymphoma	88	83	0.0	0.0	2.4	97.6
	T-cell non-Hodgkin’s lymphoma	25	21	0.0	23.8	23.8	52.4
	Mantle cell lymphoma	18	17	0.0	0.0	0.0	100.0
	Marginal zone lymphoma	16	15	0.0	6.7	0.0	93.3
	Diffuse large B-cell lymphoma (DLBCL) in the testis	16	15	0.0	33.3	6.7	60.0
	Burkitt lymphoma	5	3	0.0	0.0	0.0	100.0
Tumors of soft tissue and bone	Granular cell tumor	23	15	6.7	0.0	20.0	73.3
	Leiomyoma	50	36	8.3	2.8	16.7	72.2
	Leiomyosarcoma	94	74	5.4	17.6	23.0	54.1
	Liposarcoma	96	73	6.8	11.0	21.9	60.3
	Malignant peripheral nerve sheath tumor (MPNST)	15	12	0.0	16.7	25.0	58.3
	Myofibrosarcoma	26	25	8.0	16.0	48.0	28.0
	Angiosarcoma	42	27	3.7	7.4	22.2	66.7
	Angiomyolipoma	91	76	3.9	6.6	7.9	81.6
	Dermatofibrosarcoma protuberans	21	12	0.0	8.3	25.0	66.7
	Ganglioneuroma	14	11	0.0	0.0	9.1	90.9
	Kaposi sarcoma	8	4	0.0	0.0	25.0	75.0
	Neurofibroma	117	86	7.0	0.0	7.0	86.0
	Sarcoma, not otherwise specified (NOS)	74	63	9.5	12.7	19.0	58.7
	Paraganglioma	41	32	12.5	6.3	34.4	46.9
	Ewing sarcoma	23	12	8.3	8.3	25.0	58.3
	Rhabdomyosarcoma	7	6	16.7	0.0	16.7	66.7
	Schwannoma	122	102	2.9	0.0	3.9	93.1
	Synovial sarcoma	12	8	0.0	0.0	37.5	62.5
	Osteosarcoma	19	12	8.3	8.3	16.7	66.7
	Chondrosarcoma	15	7	14.3	28.6	0.0	57.1
	Rhabdoid tumor	5	4	0.0	0.0	25.0	75.0
	Solitary fibrous tumor	17	17	5.9	0.0	23.5	70.6

**Table 2:** HMGB1 immunostaining and tumor phenotype.

Tumor Entity	Pathological and Molecular Parameters		HMGB1 Immunostaining					
		n	Negative (%)	Weak (%)	Moderate (%)	Strong (%)	*p*	OR (95%CI)
Invasive breast carcinoma of no special type	pT1	143	2.8	13.3	33.6	50.3	0.0038	0.19 (0.04–0.95)
	pT2	172	10.5	10.5	40.7	38.4		
	pT3–4	33	9.1	30.3	30.3	30.3		
	G1	11	0	0	54.5	45.5	0.3749	1.48 (0.43–5.11)
	G2	192	8.3	12.5	37	42.2		
	G3	149	6.7	15.4	34.9	43		
	pN0	174	5.7	12.1	36.2	46	0.2322	0.25 (0.02–3.02)
	pN1	91	9.9	16.5	34.1	39.6		
	pN2	39	7.7	12.8	43.6	35.9		
	pN3	14	7.1	28.6	50	14.3		
Clear cell renal cell carcinoma	ISUP 1	233	12.4	6.9	17.6	63.1	<0.0001	0.16 (0.07–0.37)
	ISUP 2	348	8.3	7.5	20.1	64.1		
	ISUP 3	229	21.8	10	27.5	40.6		
	ISUP 4	62	25.8	16.1	37.1	21		
	Fuhrman 1	54	7.4	1.9	16.7	74.1	<0.0001	0.11 (0.03–0.38)
	Fuhrman 2	601	9.2	7	19.1	64.7		
	Fuhrman 3	257	21.4	9.7	26.8	42		
	Fuhrman 4	76	22.4	15.8	36.8	25		
	Thoenes 1	305	10.2	5.6	18.7	65.6	<0.0001	0.22 (0.11–0.44)
	Thoenes 2	419	17.2	10.5	26.5	45.8		
	Thoenes 3	82	23.2	15.9	28	32.9		
	UICC 1	275	10.2	5.5	21.5	62.9	<0.0001	0.14 (0.07–0.31)
	UICC 2	32	12.5	9.4	31.3	46.9		
	UICC 3	79	15.2	20.3	25.3	39.2		
	UICC 4	58	32.8	15.5	22.4	29.3		
	pT1	595	7.7	5	19.2	68.1	<0.0001	0.18 (0.11–0.27)
	pT2	113	14.2	11.5	29.2	45.1		
	pT3–4	285	23.9	13	26	37.2		
	pN0	147	16.3	9.5	26.5	47.6	0.0108	0.15 (0.03–0.61)
	pN+	23	30.4	13	43.5	13		
	pM0	93	12.9	7.5	30.1	49.5	0.0413	0.36 (0.15–0.85)
	pM+	78	24.4	15.4	26.9	33.3		
Urothelial bladder carcinoma	pTa G2 low	78	0	1.3	3.8	94.9	0.0078	64.83 (5.83–720.42)
	pTa G2 high	68	0	0	4.4	95.6		
	pTa G3	81	1.2	2.5	18.5	77.8		
	pT2	118	1.7	12.7	27.1	58.5	0.2152	0.54 (0.11–2.75)
	pT3	206	2.9	9.7	33.5	53.9		
	pT4	101	0	6.9	33.7	59.4		
	G2	22	0	13.6	18.2	68.2	0.3532 *	1.15 (0.32–4.15)
	G3	403	2	9.7	32.5	55.8		
	pN0	233	1.7	9.9	34.3	54.1	0.6253 *	0.78 (0.19–3.24)
	pN+	164	2.4	8.5	29.3	59.8		
	L0	166	1.2	12	34.3	52.4	0.2492 *	0.53 (0.09–2.96)
	L1	155	2.6	7.1	30.3	60		
	V0	234	1.7	10.3	35.5	52.6	0.4817 *	0.73 (0.13–4.12)
	V1	75	2.7	9.3	26.7	61.3		
Endometrioid endometrial carcinoma	pT1	103	0	6.8	48.5	44.7	0.1693	0.91 (0.21–3.9)
	pT2	24	4.2	16.7	25	54.2		
	pT3–4	35	0	8.6	40	51.4		
	pN0	49	0	6.1	42.9	51	0.1588	0.27 (0.05–1.44)
	pN+	29	3.4	13.8	51.7	31		
Serous carcinoma of the ovary	pT1	29	3.4	24.1	27.6	44.8	0.1841	2.24 (0.82–6.1)
	pT2	41	0	19.5	31.7	48.8		
	pT3	244	0	12.3	36.5	51.2		
	pN0	78	1.3	24.4	35.9	38.5	0.0213	2.76 (1.3–5.86)
	pN+	156	0	12.8	31.4	55.8		
Adenocarcinoma of the colorectum	pT1	76	0	1.3	14.5	84.2	0.1017	0.22 (0.03–1.73)
	pT2	389	0	2.8	18.3	78.9		
	pT3	1127	0.4	4.4	21.2	74		
	pT4	397	0	5	22.2	72.8		
	pN0	1023	0.4	3.4	22.3	73.9	0.0137	0.7 (0.45–1.09)
	pN+	961	0	5	19.3	75.8		
	V0	1424	0.2	4.4	20.8	74.6	0.602	1.04 (0.11–9.68)
	V1	530	0.2	3.4	19.1	77.4		
	L0	626	0.2	4.5	18.7	76.7	0.4645	0.96 (0.12–8.09)
	L1	1332	0.2	4.1	21.8	74		
	Left side	1111	0.2	2.2	21	76.7	0.0129	0.66 (0.07–6.13)
	Right side	397	0.3	5.5	18.4	75.8		
	MMR-deficient	73	0	5.5	30.1	64.4	0.0167	2.87 (0.96–8.58)
	MMR-proficient	1023	0.1	2.4	16.8	80.6		
	RAS mutation	348	0.6	2	20.7	76.7	0.1566	0.96 (0.36–2.55)
	RAS wildtype	453	0	2.2	17	80.8		
	BRAF V600E mutation	21	0	0	38.1	61.9	0.0566	2.93 (1.08–7.94)
	BRAF wildtype	126	0	4	16.7	79.4		
Adenocarcinoma of the pancreas	pT1	8	0	0	25	75	0.6571	0.48 (0.07–3.09)
	pT2	49	0	10.2	26.5	63.3		
	pT3	285	0.7	5.6	34	59.6		
	pT4	19	5.3	5.3	36.8	52.6		
	G1	11	0	0	27.3	72.7	0.5005	0.54 (0.13–2.22)
	G2	256	1.2	5.9	32.4	60.5		
	G3	78	0	9	37.2	53.8		
	pN0	71	0	7	35.2	57.7	0.6389	1.25 (0.44–3.6)
	pN+	289	1	5.9	32.2	60.9		
Adenocarcinoma of the stomach	pT1–2	45	2.2	15.6	35.6	46.7	0.7925	2.19 (0.13–36.73)
	pT3	104	1.9	23.1	33.7	41.3		
	pT4	101	1	14.9	37.6	46.5		
	pN0	63	1.6	14.3	31.7	52.4	0.5553	0.81 (0.08–7.98)
	pN+	187	1.6	19.3	36.9	42.2		
	MMR-deficient	30	0	13.3	43.3	43.3	0.5888	1.02 (0.31–3.35)
	MMR-proficient	220	2.3	14.5	35	48.2		
Seminomas	pT1	307	69.7	19.9	9.8	0.7	0.6961	3.59 (0.33–39.49)
	pT2	120	70	21.7	7.5	0.8		
	pT3	48	58.3	25	14.6	2.1		
	Haemangioinvasion negative	394	67	21.8	10.7	0.5	0.1706	7.95 (1.08–58.49)
	Positive	48	70.8	18.8	6.3	4.2		
	Lymphangioinvasion negative	341	67.2	21.7	10.9	0.3	0.1529	9.47 (0.99–90.33)
	Positive	105	68.6	20	8.6	2.9		
	Infiltration of the spermatic cord negative	370	70	20	9.2	0.8	0.5083	2.62 (0.26–26.27)
	Positive	52	63.5	19.2	15.4	1.9		
	Infiltration Rete Testis negative	211	69.2	19.9	10.4	0.5	0.8455	1.56 (0.14–16.91)
	Positive	241	71	19.9	8.3	0.8		
Squamous cell carcinomas of different sites **	pT1	170	1.2	10.6	30	58.2	0.1428	0.18 (0.04–0.93)
	pT2	192	3.6	15.1	33.9	47.4		
	pT3	111	3.6	13.5	28.8	54.1		
	pT4	108	5.6	20.4	24.1	50		
	G1	21	9.5	0	61.9	28.6	0.001	7.51 (1.14–49.43)
	G2	276	4.3	17.8	26.1	51.8		
	G3	177	2.3	11.3	34.5	52		
	pN0	230	4.3	13.5	27	55.2	0.3486	2.26 (0.68–7.49)
	pN+	218	1.8	15.1	30.7	52.3		

* only in pT2–4 urothelial bladder carcinomas, ** oral, pharynx, larynx, esophagus, cervix, vagina, vulva, penis, anal, and lung; abbreviation: pT: pathological tumor stage, G: grade, pN: pathological lymph node status, pM: pathological status of distant metastasis, R: resection margin status, V: venous invasion, L: lymphatic invasion, MMR: mismatch repair, ISUP: International Society of Urological Pathology, UICC: Union for International Cancer Control, OR: odds ratios are provided for the lowest vs. the highest parameter.

## Data Availability

The original contributions presented in the study are included in the article/Supplementary Material, further inquiries can be directed to the corresponding author.

## Supplementary Materials

The following supporting information can be downloaded at https://www.mdpi.com/article/10.3390/diagnostics15151974/s1, Supplementary Figure S1. Immunohistochemistry validation by comparison with an independent second antibody. The panels show a full confirmation of IHC results by an independent second HMGB1 antibody. Using HMV317, nuclear HMGB1 staining is lacking in maturing cells of the spermatogenesis in the testis (A), decreases from the basal cell layers to the top layers in squamous epithelium of the tonsil (B), is variable and often low in proximal tubuli of the kidney (C), and is lower in acinar than in basal cells of the prostate (D). Using clone EPR3507, a comparable staining was seen in the testis (E), tonsil (F), kidney (G), and the prostate (H). Supplementary Figure S2: Further samples of HMGB1 immunostaining observed in different tumor types. (A–E): Tumors with complete loss of HMGB1 expression, including (A) an endometrioid carcinoma of the ovary, (B) clear cell renal cell carcinoma, (C) clear cell carcinoma of the ovary, (D) adenocarcinoma of the colon, and (E) lobular carcinoma of the breast. (F–J): HMGB1-positive tumors, including (F) a breast cancer of no special type, (G) squamous cell carcinoma of the anal canal, (H) neurofibroma, (I) pancreatic adenocarcinoma, and (J) a muscle-invasive urinary bladder cancer. Supplementary Figure S3: Examples of HMGB1 immunostaining scores. (A) Score 0: Absence of HMGB1 staining in a clear cell carcinoma of the ovary, (B) Score 1+: weak HMGB1 staining in a case of adenocarcinoma of the pancreas, (C) Score 2+: HMGB1 staining of moderate intensity in an adenocarcinoma of the colon, and (D) Score 3: strong HMGB1 staining in adenocarcinoma of the colon.
