# Supplementary material for: HMGB1 Deficiency Occurs in a Broad Range of Human Cancers and Is Often Associated with Unfavorable Tumor Phenotype
Source: Diagnostics (Basel). 2025 Aug 6;15(15):1974. doi: 10.3390/diagnostics15151974 (PMC12346012; doi:10.3390/diagnostics15151974)
Supplement: Supplementary file 1 [file diagnostics-15-01974-s001.zip › Suppl Figure S2 HGMB1_R1_rs_hub.pdf]

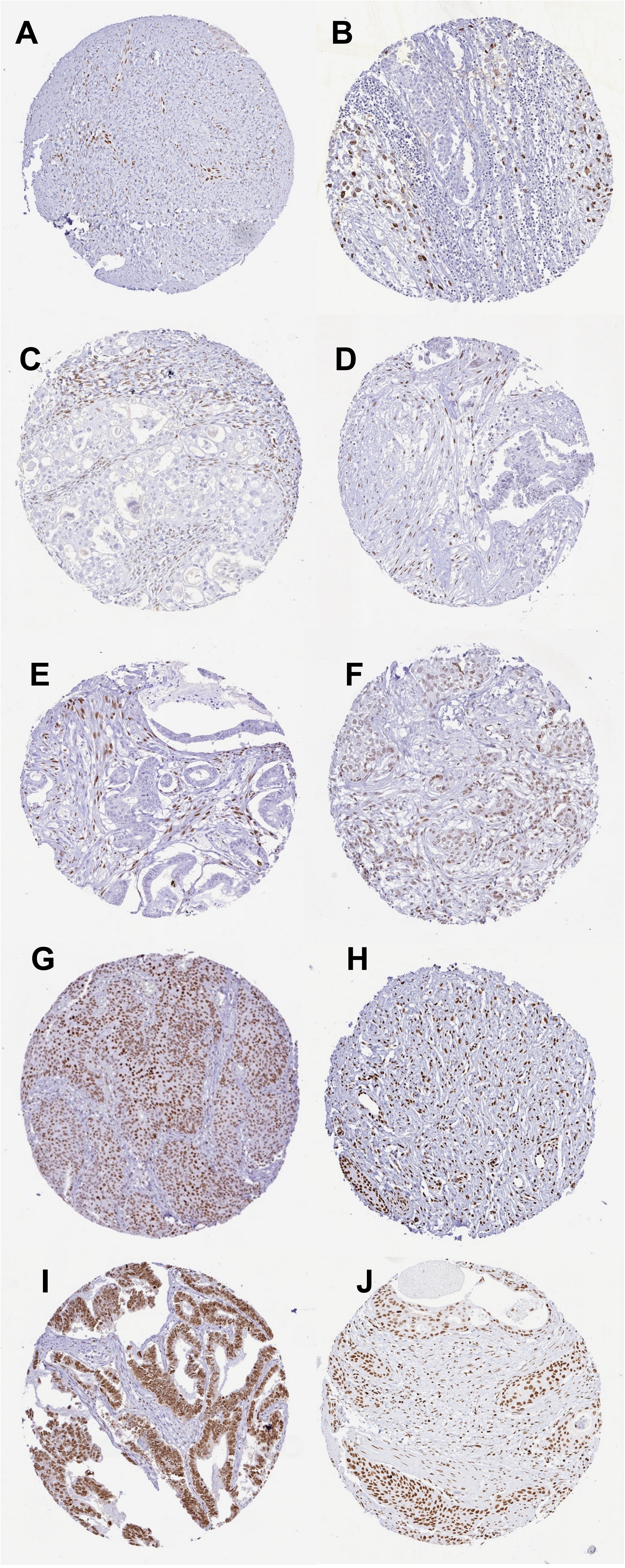

Supplementary Figure 2: Further samples of HMGB1 immunostaining observed in different tumor types. A-E): Tumors with complete loss of HMGB1 expression, including A) an endometrioid carcinoma of the ovary , B) clear cell renal cell carcinoma , C) clear cell carcinoma of the ovary, D) adenocarcinoma of the colon and E) lobular carcinoma of the breast. F-J): HMGB1 – positive tumors, including F) a breast cancer of no special type, G) squamous cell carcinoma of the anal canal, H) neurofibroma, I) pancreatic adenocarcinoma and J) a muscle invasive urinary bladder cancer.
