# Supplementary material for: HMGB1 Deficiency Occurs in a Broad Range of Human Cancers and Is Often Associated with Unfavorable Tumor Phenotype
Source: Diagnostics (Basel). 2025 Aug 6;15(15):1974. doi: 10.3390/diagnostics15151974 (PMC12346012; doi:10.3390/diagnostics15151974)
Supplement: Supplementary file 1 [file diagnostics-15-01974-s001.zip › Suppl Figure S3_HGMB1_R1.pdf]

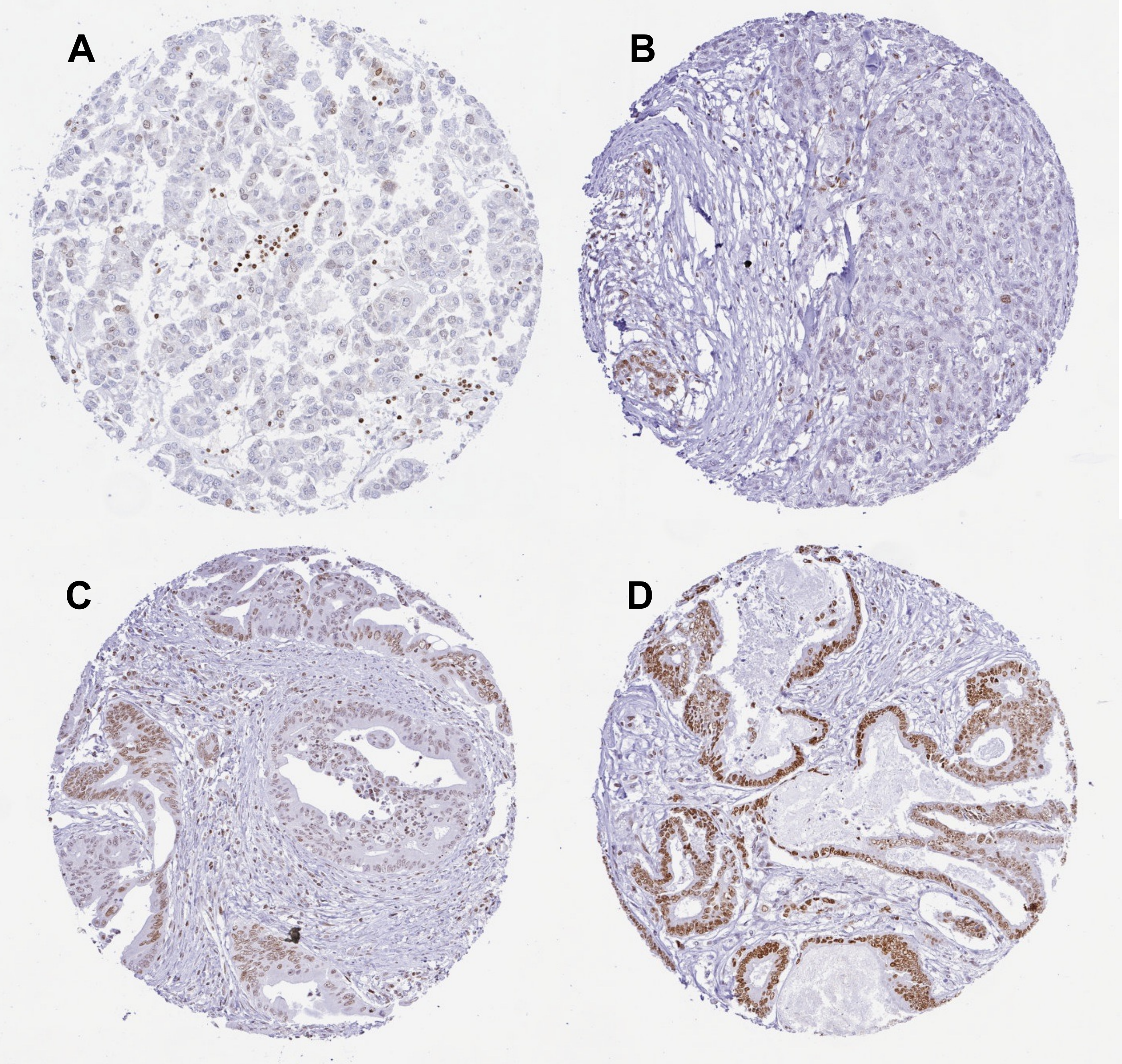

Supplementary Figure 3: Examples of HMGB1 immunostaining scores. A) Score 0: Absence of HMGB1 staining in a clear cell carcinoma of the ovary, B) Score 1+: weak HMGB1 staining in a case of adenocarcinoma of the pancreas, C) Score 2+: HMGB1 staining of moderate intensity in an adenocarcinoma of the colon, and D) Score 3: strong HMGB1 staining in adenocarcinoma of the colon.
