# Supplementary figures and images for: HMGB1 Deficiency Occurs in a Broad Range of Human Cancers and Is Often Associated with Unfavorable Tumor Phenotype
Source: Diagnostics (Basel). 2025 Aug 6;15(15):1974. doi: 10.3390/diagnostics15151974 (PMC12346012; doi:10.3390/diagnostics15151974)

A

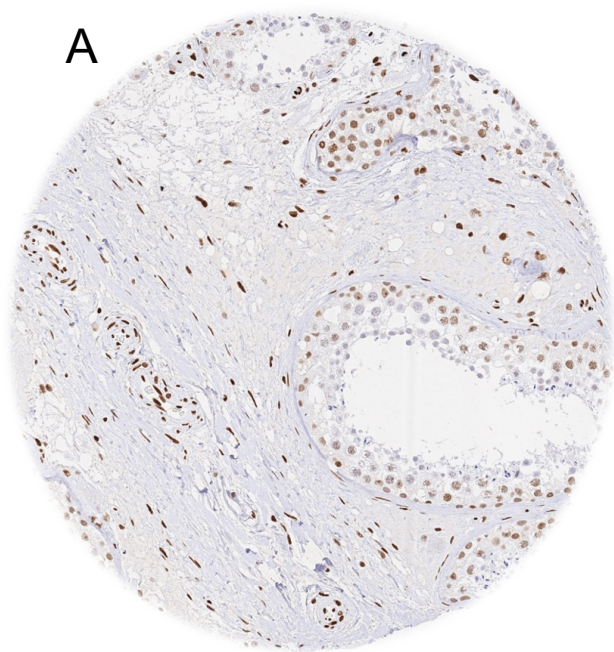

B

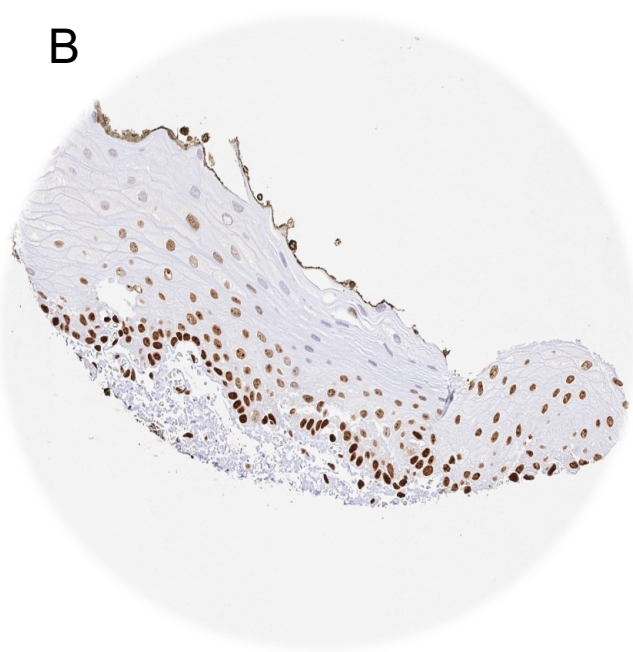

C

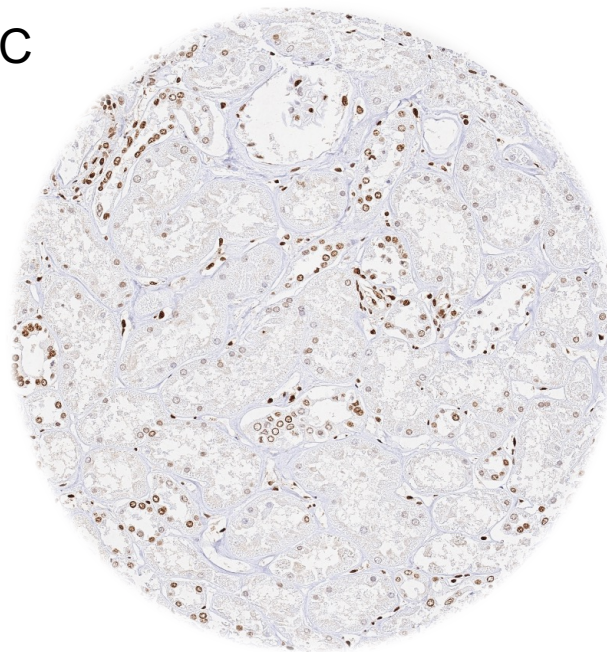

D

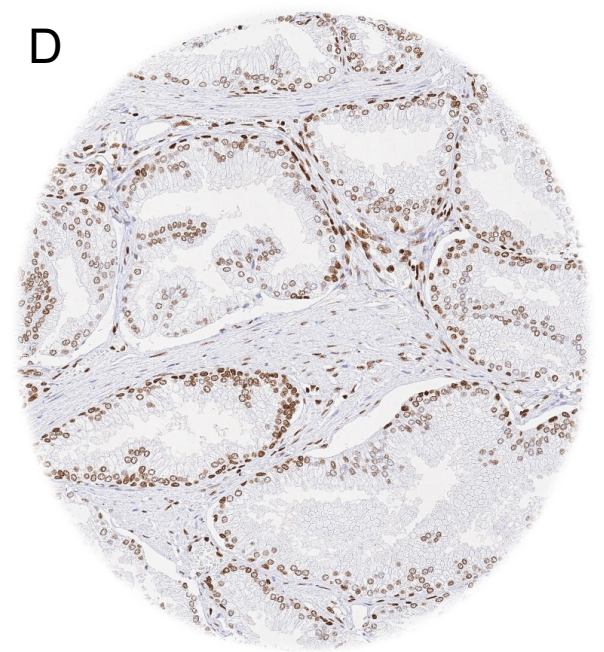

E

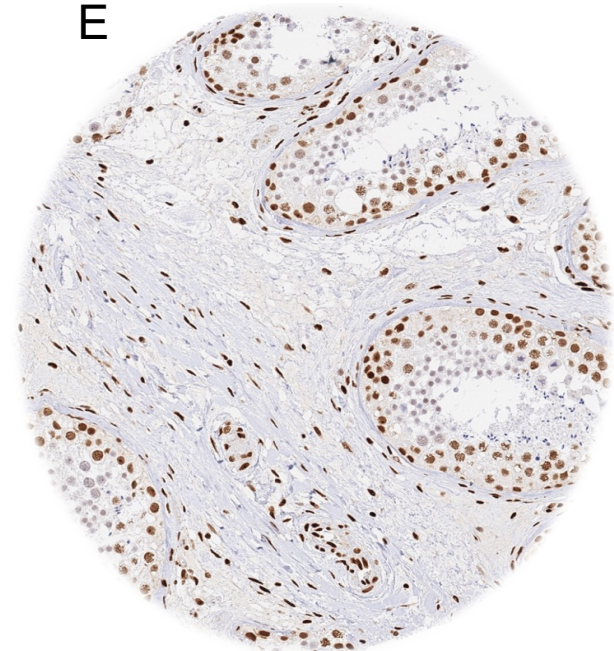

F

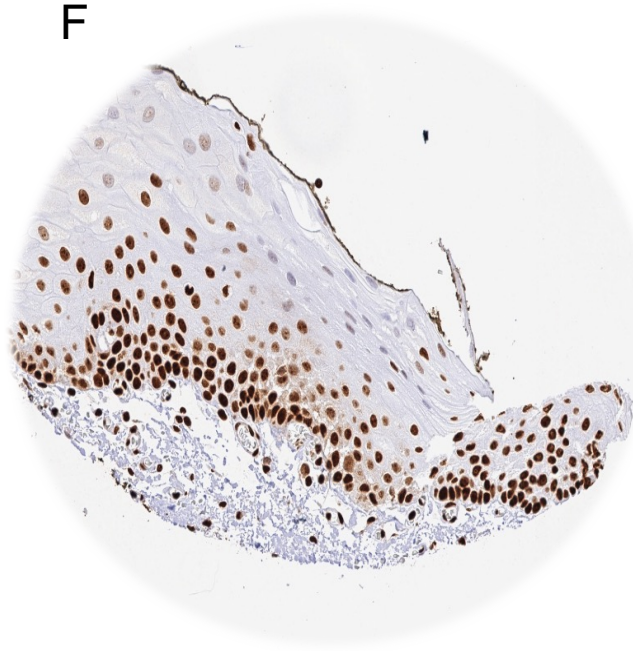

G

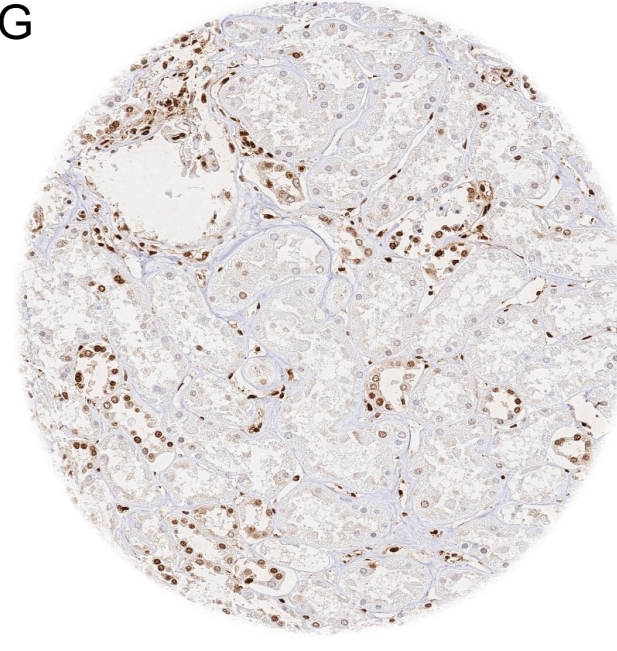

H

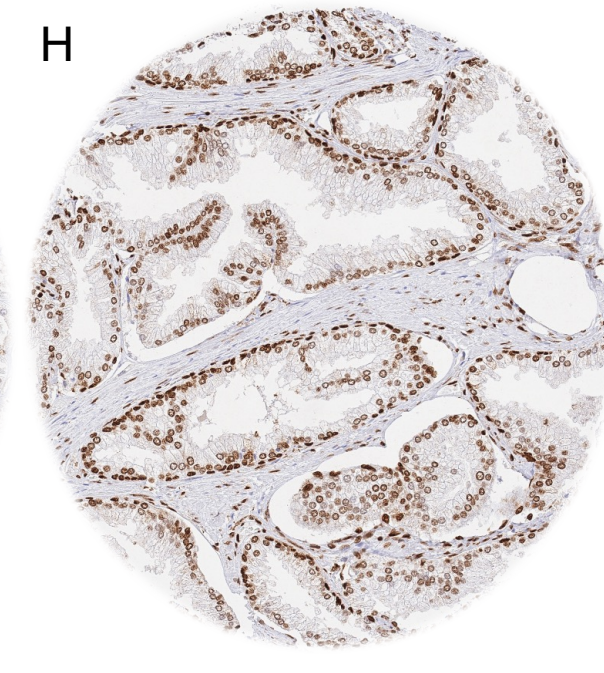

Supplement: Supplementary file 1 [file diagnostics-15-01974-s001.zip › Suppl Figure S1_HMGB1.pdf]
